# Insights from the Complete Chloroplast Genome into the Evolution of *Sesamum indicum* L

**DOI:** 10.1371/journal.pone.0080508

**Published:** 2013-11-26

**Authors:** Haiyang Zhang, Chun Li, Hongmei Miao, Songjin Xiong

**Affiliations:** 1 Henan Sesame Research Center, Henan Academy of Agricultural Sciences, Zhengzhou, People's Republic of China; 2 TEDA School of Biological Sciences and Biotechnology, Nankai University, Tianjin, People's Republic of China; Beijing Institute of Genomics, China

## Abstract

Sesame (*Sesamum indicum* L.) is one of the oldest oilseed crops. In order to investigate the evolutionary characters according to the Sesame Genome Project, apart from sequencing its nuclear genome, we sequenced the complete chloroplast genome of *S. indicum* cv. Yuzhi 11 (white seeded) using Illumina and 454 sequencing. Comparisons of chloroplast genomes between *S. indicum* and the 18 other higher plants were then analyzed. The chloroplast genome of cv. Yuzhi 11 contains 153,338 bp and a total of 114 unique genes (KC569603). The number of chloroplast genes in sesame is the same as that in *Nicotiana tabacum*, *Vitis vinifera* and *Platanus occidentalis*. The variation in the length of the large single-copy (LSC) regions and inverted repeats (IR) in sesame compared to 18 other higher plant species was the main contributor to size variation in the cp genome in these species. The 77 functional chloroplast genes, except for *ycf1* and *ycf2*, were highly conserved. The deletion of the cp *ycf1* gene sequence in cp genomes may be due either to its transfer to the nuclear genome, as has occurred in sesame, or direct deletion, as has occurred in *Panax ginseng* and *Cucumis sativus*. The sesame *ycf2* gene is only 5,721 bp in length and has lost about 1,179 bp. Nucleotides 1–585 of *ycf2* when queried in BLAST had hits in the sesame draft genome. Five repeats (R10, R12, R13, R14 and R17) were unique to the sesame chloroplast genome. We also found that IR contraction/expansion in the cp genome alters its rate of evolution. Chloroplast genes and repeats display the signature of convergent evolution in sesame and other species. These findings provide a foundation for further investigation of cp genome evolution in *Sesamum* and other higher plants.

## Introduction

Sesame (*Sesamum indicum* L., 2n = 26), which belongs to the Pedaliaceae family, is one of the oldest and most important oilseed crops [1]. The history of its cultivation can be traced back to 3050–3500 BC in the Harappa Valley of the Indian subcontinent [2]. Currently sesame is grown worldwide in tropical and subtropical regions with a total area of about 7.8 million hectares, and annual production of 3.84 million tons (2010, FAO). Sesame as an oilseed crop has one of the highest oil-contents at 50–60% [1], [3] and is mainly used for oil and food [4], [5].

*S. indicum* is in the asterids clade of the core eudicotyledons in Angiosperm Phylogeny Group 2 (APG 2) (Angiosperm Phylogeny Group, 2003). Compared with 36 plant species from 19 families using publically-available genomic datasets (NCBI), *Sesamum* is closely related to members of the Solanaceae and Phrymaceae families, but distant to the other oil crops such as soybean (*Glycine max*), castor (*Ricinus communis*) and rape (*Brassica rapa*) [6]. The chloroplast (cp) genome sequence of *S. indicum* cv. Ansanggae (a black-seeded cultivar) was published recently [7]. Its phylogenetic position suggests that *Sesamum* is a sister genus to the *Olea* and *Jasminum* (Oleaceae family) and is located in the core lineage of the Lamiales family [7]. However, the origin and phylogeny of *Sesamum* still requires clarification [1], [8]. The evolutionary process and relationship between sesame and other oil crops has not been explored using genomic data.

The chloroplast is a vital plastid in plants and algae, containing all the enzymatic machinery required for plant photosynthesis and related genomic information [9], [10]. It is regarded as one of the most important indices for comparative evolutionary analysis and molecular taxonomy, as the cp genome is relatively conservative and independent of the nuclear genome [11]. In most plants, the circular cp genome is 120–160 Kb, and usually contains about 4 rRNAs, 30 tRNAs and 80 protein-coding genes related to photosynthesis or gene expression [12], [13]. The cp genome is present at high copy number and has been used in genetic modification and crop breeding studies [14]–[18]. To date, more than one hundred cp genomes have been sequenced (Chloroplast Genome DB, http://chloroplast.cbio.psu.edu).

As part of the ongoing Sesame Genome Project (www.sesamum.org), we have used Illumina and 454 sequencing to sequence and assemble the complete cp genome of cv. Yuzhi 11. We have also performed a comparative evolutionary analysis of cp genomes between sesame and other major crops using publically-available genomic datasets, thus revealing some features of sesame evolution.

## Materials and Methods

### Plant material and isolation of sesame cp genome DNA

Yuzhi 11 (white-seeded), a major Chinese domestic cultivar and the cultivar we used to sequence the sesame genome [6], [19], was grown at Yuanyang Experimental Station, Henan Academy of Agricultural Sciences (HAAS) in 2011. Approximately 100 g young leaf tissue was harvested for extraction of cp genome DNA.

Intact chloroplasts of *S. indicum* were collected by sucrose density gradient centrifugation [20]. Fresh leaves were fully homogenized in chloroplast isolation buffer (0.3 M Sorbitol, 5 mM EDTA, 5 mM MgC1_2_, 1 mM DTT, 5 mM KH_2_PO_4_, 5 mM K_2_HPO_4_, 10 mM 2-Mercaptoethanol and 2 mM Ascorbic acid) at 0°C. In order to remove the cell wall debris and unbroken cells, the homogenate was gently filtered through 8 layers of cheesecloth and then centrifuged at 200 g for 5 min at 4°C before resuspending in chloroplast isolation buffer. Intact chloroplasts were purified by further sucrose density gradient centrifugation at 2,500 g for 15 min and then at 3,500 g for 30 min. Chloroplast genome DNA was isolated using chloroplast lysis buffer (10 mM Tris, 2% sodium dodecyl sulphate and 0.4% sodium N-lauroylsarcosine). Proteinase K and RNase were added to remove all protein and RNA from the cp DNA solution. The quality of cp genome DNA was analyzed by pulsed-field gel electrophoresis (PFGE). 20 µg of cp genome DNA was prepared for constructing a Solexa library, and an equal amount of DNA was reserved for 454 sequencing and gap-filling.

### High-throughput sequencing

We sequenced the sesame cp genome using both Illumina and 454 sequencing. High-throughput sequencing of the *S. indicum* cp genome was first carried out on an Illumina GA IIx platform. Paired-end and mate-pair libraries with insert sizes of 500 bp and 3 Kb, respectively, were constructed using proprietary reagents according to the manufacturer's recommended protocols (https://icom.illumina.com/). Paired-end and mate-pair libraries were denatured and then diluted in hybridization buffer before loading into an Illumina GA flowcell. 101×2 cycle sequencing was performed according to the manufacturer's instructions. To accurately assign the repeat regions to the cp genome, Roche 454 reads from paired-end (PE) libraries with an insert size of 8 Kb were also used. The Roche 454 reads were generated in the Sesame Genome Project (www.sesamum.org) [6], and ranged from 64 to 1,199 bp in length. Constructing 8 Kb PE library and 454 sequencing were performed according to the protocols described by Jarvie and Harkins [21].

### Cp genome assembly

Raw reads generated by Illumina-Solexa GAIIx were pre-processed using SolexaQA [22]. Low quality bases (Q<13) were trimmed and all reads shorter than 25 bp were discarded. Trimmed reads were re-paired with an in-house perl script. To efficiently assemble the cp genome, a method as below was performed (see Figure S1). All quality-filtered paired reads were mapped against the cp genomes of *Ageratina adenophora* (NCBI: NC_015621.1) and *Olea europaea* (NCBI: NC_015623) using the BWA-SW algorithm and the defaulted parameters [23]. The yielded reads were definitely from the sesame cp genome. Then all mapped reads and their mates were *de novo* assembled using Velvet [24]. Subsequently, Roche 454 raw reads of sesame nuclear and cp genomes were aligned to the contigs generated in Velvet using GS Reference Mapper (454 Life Science). The mapped Roche 454 reads were definitely from the sesame cp genome. GS De Novo Assembler (v2.6) was used to assemble the extracted Roche 454 reads, and the draft genome was assembled. Potential gaps and the IR (inverted repeat, a collapsed consensus of IRa and IRb), LSC (large single-copy) and SSC (small single-copy) region of the draft genome were identified after aligned to the cp genome of *A. adenophora* with BLAST. PCR walking and capillary electrophoresis sequencing (ABI 3730xl sequencer) were performed to fill the gaps and to verify the junctions between the single-copy and the IRs regions. The primers used in this step were developed using Consed (v 20.0). After gap-filling, Illumina-Solexa reads and the BWA were used to verify the bases and to correct potential assembly errors.

### Bioinformatics analysis

The *S. indicum* cp genome was annotated with DOGMA (Dual Organellar GenoMe Annotator) [25]. A circular map of the sesame cp genome was drawn using Circos [26]. Repeats and Inverted Repeats (IR) within the sesame cp genome were identified using REPuter, using criteria of length cutoff ≥30 bp and sequence identity ≥90% [27]. Protein-coding and noncoding sequences from *S. indiucm* and the 18 species were aligned using MEGA 5.0 with the MUSCLE-codon (Multiple Sequence Caparison by Log-Expectation) and MUSCLE model, respectively [28]. Sequence alignments at whole cp genome level were also performed in MEGA 5.0 with MUSCLE model. In all the above sequence alignments, default settings were used. *K*a (nonsynonymous substitution rates), *K*s (synonymous substitution rates) and their ratio were calculated by the *K*a*K*s_Calculator program [29] using MA (Model Averaging).

## Results

### Sequencing of the complete cp genome and its structure in sesame

**Figure 1:**
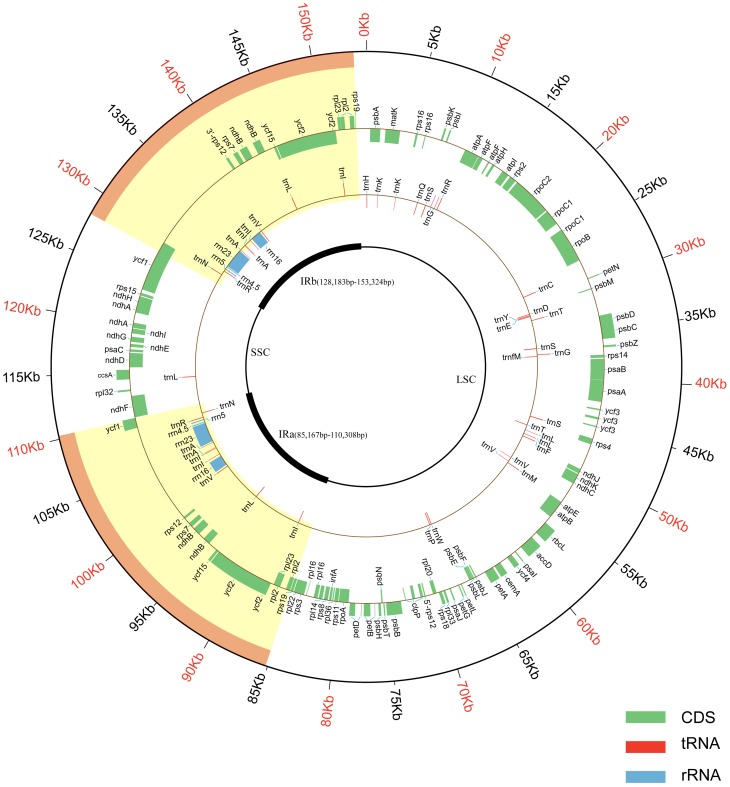
Sesame cv. Yuzhi 11 cp genome map. The two thick lines in the inner circle represent the IRa and IRb Inverted Repeat sequences which separate the LSC and SSC regions. Genes of the inner circle are transcribed clockwise, while those of the outer circle are transcribed anti-clockwise.

After Illumina and Roche 454 sequencing, the Illumina and 454 raw reads were mapped using Velvet and GS *De Novo* Assembler, respectively. The mapped reads gave a coverage of approximate 218× cp genome. Using the 454 mapped reads, the draft genome was assembled into four scaffolds ranging from 10,567 to 65,797 bp in length. The draft genome covered 99% of the genome part and contained the LSC, SSC and one IR region. After gap filling, single-copy and IRs region identifying and sequence verifying, the complete cp genome of *S. indicum* cv. Yuzhi 11 was formed. The sesame cp genome is a circular molecule containing a total of 153,338 base pairs (GenBank accession no. KC569603) (Figure 1). The three scaffolds of this cp genome were found to contain the inverted repeat (IR, a collapsed consensus of IRa and IRb, 25,142 bp), large single-copy (LSC, 85,180 bp) and small single-copy (SSC, 17,874 bp) regions. A total of 114 unique genes, encoding 80 proteins, 30 tRNA and 4 rRNA, were identified in the cp genome (Yuzhi 11 genotype). As shown in Figure 1, two copies of 8 protein-coding genes, 7 tRNA and 4 rRNA genes are present in the IR region. Of the 153, 338 bp, protein-coding genes, tRNA genes and rRNA genes occupy 50.44% 1.84% and 5.90%, respectively. There are 18 intron-containing genes in the cp genome, of which 16 contain one intron, and 2 (*ycf3* and *clpP*) have two introns. The overall AT content is 61.8%. The ratio of AT content in protein-coding genes, tRNA and rRNA sequences is 61.78%, 47.34%, and 44.73% respectively.

To evaluate the degree of conservation of the sesame cp genome, we compared the cp genomes of cv. Yuzhi 11 with that of cv. Ansanggae (NC_016433.2) (Table 1). Results showed that there are only 14 differences within the nucleotide sequences of homopolymers. The number of repeat nucleotides in the 14 homopolymers of the cv. Ansanggae cp genome had uniformly one less base than homopolymers from the cv. Yuzhi 11 cp genome.

**Table 1:** Variation in the cp genome sequence between Yuzhi 11 (KC569603) and Ansanggae (NC_016433.2).

	Sequence position (bp)	Nucleotide number^1^	
		KC569603	NC_016433.2
1	8,564	A(11)	A(10)
2	16,670	T(10)	T(9)
3	16,681	A(9)	A(8)
4	29,725	T(10)	T(9)
5	46,882	T(10)	T(9)
6	47,677	A(10)	A(9)
7	60,482	A(9)	A(8)
8	61,778	A(10)	A(9)
9	82,858	T(10)	T(9)
10	82,924	A(10)	A(9)
11	99,217	T(9)	T(8)
12	112,950	T(10)	T(9)
13	117,198	A(10)	A(9)
14	139,266	A(9)	A(8)

Note: ^1^ data in brackets indicates the number of repetitions of specific nucleotides in the two cp genome sequences.

Then comparisons of cp genome sequence and structure were analyzed between *S. indicum* and the 18 species presenting the available nuclear genome sequences (listed in Table 2). The number of cp genes in sesame is the same as that in *Nicotiana tabacum*, *Vitis vinifera*, *Platanus occidentalis* (NCBI data). Among the 19 cp genomes examined, *infA*, *infA* and *rpl22*, *infA*, and *infA*, were missing in *Arabidopsis thaliana*, *Glycine max*, *Brassica napus* and *Mangifera indica*, respectively. Gene order in the sesame cp genome is highly conserved, being similar to that of *N. tabacum*, *A. thaliana*, *P. occidentalis* and *B. napus*, but different from that of *G. max*, *Helianthus annuus* and *Gossypium hirsutum* (NCBI data).

**Table 2:** Comparison of cp genome size between sesame and 18 other species.

Species	Accession no.	Cp genome sequence				
		Length (bp)	Length variation (bp)	LSC variation (bp)	SSC variation (bp)	IR variation (bp)
*Sesamum indicum*	KC569603	153,338	-	-	-	-
*Vitis vinifera*	NC_007957	160,928	7,590	3,969	1,199	1,211
*Mangifera indica*	NC_008359	158,484	5,146	2,206	1,868	536
*Arabidopsis thaliana*	NC_000932	154,478	1,140	−1,010	−94	1,122
*Brassica napus*	NC_016734	152,860	−478	−2,150	−114	893
*Glycine max*	NC_007942	152,218	−1,120	−2,005	21	432
*Cucumis sativus*	DQ119058	155,527	2,189	1,699	400	45
*Gossypium hirsutum*	NC_007944	160,301	6,963	3,636	2,407	460
*Ricinus communis*	NC_016736	163,161	9,823	4,471	942	2,205
*Platanus occidentalis*	NC_008335	161,791	8,453	6,970	1,635	−76
*Helianthus annuus*	NC_007977	151,104	−2,234	−1,650	434	−509
*Nicotiana tabacum*	NC_001879	155,943	2,605	1,506	697	201
*Olea europaea*	GU931818	155,889	2,551	1,434	−83	600
*Coffea arabica*	NC_008535	155,189	1,851	−14	333	766
*Panax ginseng*	NC_006290	156,318	2,980	926	196	929
*Triticum aestivum*	NC_002762	134,545	−18,793	−4,831	−5,084	−4,439
*Oryza sativa*	NC_001320	134,525	−18,813	−4,588	−5,539	−4,343
*Zea mays*	NC_001666	140,387	−12,951	−2,825	−5,338	−2,394
*Sorghum bicolor*	NC_008602	140,754	−12,584	−1,447	−5,371	−2,883

Note: Data indicates the difference in the length of cp genomes relative to *S. indicum*. The length of the sesame cp genome is ranked 12^th^ among the 19 species.

### Variation in the length of cp genomes between sesame and 18 other plant species

In order to clarify the evolutionary position of the sesame cp genome among higher plants, we conducted a phylogenetic analysis using data from 19 cp genomes (Table 2). Results were consistent with previous reports (Figure S2) [6]. The size of the sesame cp genome was smaller than that of 11 species such as *V. vinifera*, *A. thaliana*, *G. hirsutum* and *N. tabacum*, but larger than that of 7 species, i.e., *B. napus*, *G. max*, *H. annuus* and four *Poaceae* species (Table 2). The lengths of the LSC and IRs in sesame differed from those in the other 18 species and contributed to the variation of cp genome size. For example, the difference in the size of the sesame and *Panax ginseng* cp genomes was 2,980 bp, differences in the lengths of LSC and IR sequences contributing 926 bp and 1858 bp, respectively. Differences in the size of the sesame and *N. tabacum* cp genomes was 2,605 bp, the LSC and IR sequences contributing 1,506 bp and 402 bp, respectively. Variation in the length of IRs had a large effect on cp genomic evolution in *A. thaliana*, *Coffea arabica*, *P. ginseng* and the *Poaceae*.

**Figure 2:**
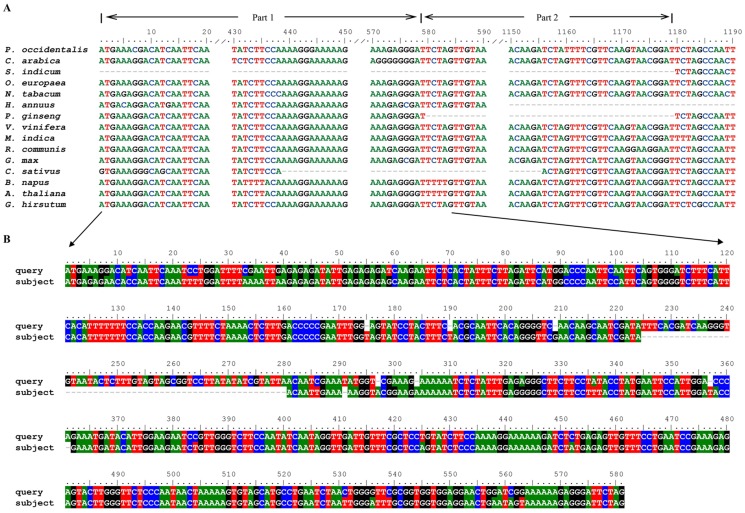
Multiple sequence alignment of *ycf2* genes in 15 species. **A**: Multiple sequence alignment of *ycf2* (1–1,190 bp). **B**: BLAST results for *ycf2* (1–584 bp) using *O. europaea* cp genome sequences and sesame genome data. 1–47 bp, 48–221 bp, 278–486 bp and 487–585 bp of the subject sequences were located in different scaffolds of the sesame draft genome.

We also compared the protein-coding sequences of the 19 cp genomes. The length of all 77 functional cp genes, except for *ycf1* and *ycf2*, was highly conserved. Multiple sequence alignments were performed on the length variation of *ycf1* and *ycf2*. While *ycf2* genes were lost in the cp genomes of four grass species, the length of the *ycf2* gene in the 14 species varied between 5, 967 bp (*Cucumis sativus*) and 6,903 bp (*V. vinifera* (Figure 2A and Figure S2)). However, the *ycf2* gene in the sesame cp genome was observed as only 5,721 bp in length and a fragment of about 1,179 bp was lost. In order to trace the missing sequence, the 1–1,179 bp region of the *ycf2* gene of *O. europaea* was selected as a reference to screen the sesame genome sequence database (about 10× coverage) (www.sesamum.org) (Figure 2B and Figure S3). BLAST results showed that nucleotides 1–585 in the query had hits in the sesame draft genome, while nucleotides 586–1,179 could not be found. Multiple sequence alignments from the 15 species showed that the sesame *ycf1* gene was shorter than those from 11 species, and the same fragments in three species, i.e., *A. thalianan*, *H. annuus* and *B. napus* were evidently lost (Figure S4). Screening results of the sesame *ycf1* gene indicated that the 1–1,000 bp fragment had highly similar hits (identities >90%) in the sesame draft genome as a query.

### Comparisons of repeats in cp genome between sesame and 18 other plant species

**Figure 3:**
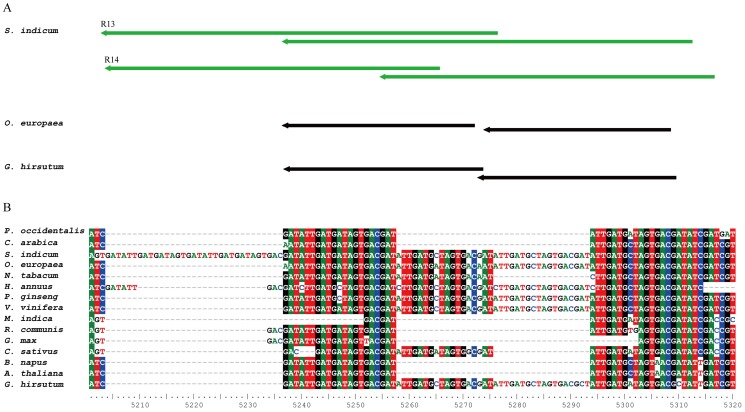
Relative locations and multiple alignments of Repeats 13 and 14 between 15 species. **A**: Relative locations of Repeats 13 and 14 in *S. indicum*, *O. europaea* and *G. hirsutum.* **B**: multiple alignments of Repeats 13 and 14 in *ycf2* genes between 15 species.

Most repeats in the cp genome are present in the introns or exons of genes. Seventeen forward and inverted repeats (≥30 bp) were identified in the sesame cp genome (Table 3). Of these repeats, R3, R13 and R14 were over 40 bp in length, while the other repeats were 30–40 bp in length. To determine their evolutionary characteristics, we used BLAST to compare the 17 repeats in the sesame cp genome with 18 other species (Table 4). The 17 repeats were roughly divided into 4 groups according to their level of conservation. Group 1 consisted of five highly conserved repeats that were present in nearly all monocots and dicots, while group 4 consisted of seven repeats that were detected only in one or a few species and had low conservation. Notably, repeats R10, R12, R13, R14 and R17 were unique to the sesame cp genome, and had no hits in other species. Furthermore, multiple sequence alignment showed that specificity of R13 and R14 in sesame is due to extension of shorter ancestral repeats (Figure 3).

**Table 3:** Distribution of interspersed and palindromic repeat sequences in the sesame cp genome.

Repeat name	Repeat number	Repeat size (bp)	Start site (bp)	End site (bp)	Repeat type*	Repeat distance	Region location	Located gene position
R1	3	32	8,593	8,624	F	−3	LSC	tRNAS-GCU-exon
			36,037	36,068		0		tRNAS-UGA-exon
			45,919	45,948	P			tRNAS-GGA-exon
R2	2	30	10,118	10,147	F	−3	LSC	tRNAG-UCC-exon2
			36,985	37,014				tRNAG-UCC-exon
R3	2	30	13,674	13,703	P	−2	LSC	atpF-atpH
			13,674	13,703				
R4	2	30	30,182	30,211	P	−1	LSC	petN-psbM
			30,224	30,253				petN-psbM
R5	2	30	36,039	36,068	P	−3	LSC	tRNAS-UGA-exon
			45,919	45,948				tRNAS-GGA-exon
R6	3	39	44,206	44,244	F	−2	LSC	ycf3-intron
			98,842	98,880				IR: rps12-tRNAV-GAC
			120,311	120,349	F	−2		SSC: ndhA-intron
R7	2	30	44,218	44,247	F	−3	LSC	ycf3-intron
			98,854	98,883				IR: rps12-tRNAV-GAC
R8	2	31	55,698	55,728	P	−3	LSC	atpB-rbcL
			66,925	66,955				psbE-petL
R9	2	44	75,458	75,501	P	−2	LSC	psbT-psbN
			75,458	75,501				
R10	2	30	76,292	76,321	P	−2	LSC	petB-intron
			76,292	76,321				
R11	2	30	89,382	89,411	F	−3	IR	ycf2-exon
			89,424	89,453				ycf2-exon
R12	2	30	91,785	91,814	F	−1	IR	ycf2-exon
			91,800	91,829				ycf2-exon
R13	2	77	91,813	91,889	F	−1	IR	ycf2-exon
			91,831	91,907				ycf2-exon
R14	2	63	91,813	91,875	F	−3	IR	ycf2-exon
			91,849	91,911				ycf2-exon
R15	2	30	107,686	107,715	F	−2	IR	rRNA4.5-rRNA5
			107,717	107,746				rRNA4.5-rRNA6
R16	2	30	110,051	110,080	F	0	IR	ycf1
			110,072	110,101				ycf1
R17	2	30	115,430	115,459	P	−3	SSC	ccsA-ndhD
			115,466	115,495				ccsA-ndhD

*Of the repeat types in the sesame cp genomes, F indicates Forward repeats and P indicates Palindromic repeats.

**Table 4:** BLAST results for repeat sequences among the 18 species.

Repeat name	Group no.	*Z. mays*	*S. bicolor*	*T. aestivum*	*O. sativa*	*V. vinifera*	*M. indica*	*G. max*	*R. communis*	*A. thaliana*	*B. napus*	*G. hirsutum*	*C. sativus*	*C. arabica*	*H. annuus*	*O. europaea*	*P. ginseng*	*N. tabacum*	*P. occidentalis*
R1	1	1	1	1	1	2	2	1	3	1		1	1	2	1	1	1	2	1
R2	1	1	1	1	1	2	2	1	2	1	1	1	2	1	1	2	1	2	1
R3	4															1			
R4	4																	1	
R5	1	1	1	1	1	1	1	1	2	1	1	1	1	1	2	1	1	1	1
R6	1	2	2	2		3	4	2	5	1	1	2	3	4	2	1	1	2	4
R7	1	1	1	1	1	1	3	1	3		1	1		4	2	1	1	2	1
R8	3					1										1	1	1	
R9	3								2			2		2	2	2			
R10	4	-	-	-	-	-	-	-	-	-	-	-	-	-	-	-	-	-	-
R11	2					2	2	2	4	2	2	2	2	2	2	2		2	2
R12	4	-	-	-	-	-	-	-	-	-	-	-	-	-	-	-	-	-	-
R13	4	-	-	-	-	-	-	-	-	-	-	-	-	-	-	-	-	-	-
R14	4	-	-	-	-	-	-	-	-	-	-	-	-	-	-	-	-	-	-
R15	2					2	2	2	4	4	4	4	4	4	4	4	2	4	2
R16	3					2	2		4					2		2	8	2	
R17	4	-	-	-	-	-	-	-	-	-	-	-	-	-	-	-	-	-	-

Note: – represents no hits. Sesame repeat sequences were used as the query. The number in the table indicates the number of hits of a repeat in the cp genome for a given species.

### IR expansion and contraction

The locations of the LSC/IR and SSC/IR junctions are regarded as an index of cp genome evolution. To identify the impact of these junctions on sesame evolution, we screened the structures of IR expansions and contractions in sesame and 14 other species (Figure S5). In sesame and 12 other cp genomes, the border of the LSC/IR junction was located within the *rps19* gene, resulting in the formation of an *rps19* pseudogene. In the *O. europaea* and *C. sativus* cp genomes, however, *rps19* pseudogenes were not present since the LSC/IR junction border was located downstream of the *rps19* gene. The length of *rps19* pseudogenes in the 13 species ranged from 24 bp to 113 bp, with that in sesame, like *Ricinus communis* and *P. occidentalis*, being 30 bp in length. The border of the SSC/IR junction in sesame was located within the *ycf1* gene, resulting in the formation of a *ycf1* pseudogene. The length of *ycf1* pseudogenes varied between 345 bp and 1, 679 bp in the 14 species. The *ycf1* pseudogene in sesame was 1,010 bp, a similar length to that in *N. tabacum*, *O. europaea*, *R. communis*, *C. arabica*, *A. thaliana*, *B. napus* and *C. sativus*. In addition, we also investigated the evolutionary rate of the part of the sequence of the *ycf1* gene located in the IR region, since the *ycf1* gene of *P. occidentalis* was chosen as the reference for *Ka* and *Ks* estimation (Table 5). The *Ka*/*Ks* of IR region-located fragments of the *ycf1* gene were significantly lower among the 13 species than those of the full sequences.

**Table 5:** Evolutionary rate of full length *ycf1* and IR region-located fragments of the *ycf1* gene.

Species name	Full length of *ycf1* gene				IR region-located fragment of *ycf1* gene			
	*K*a	*K*s	*K*a*/K*s	Length (bp)	*K*a	*K*s	*K*a*/K*s	Length (bp)
*V. vinifera*	0.159	0.350	0.455	5,565	0.175	0.439	0.400	4,536
*M. indica*	0.219	0.507	0.433	5,439	0.258	0.714	0.362	4,455
*G. max*	0.324	0.818	0.396	5,157	0.350	0.964	0.363	4,680
*C. sativus*	0.306	0.651	0.469	5,136	0.370	1.004	0.368	4,080
*R. communis*	0.219	0.556	0.393	5,244	0.265	0.814	0.325	4,077
*A. thaliana*	0.286	0.904	0.316	5,298	0.340	1.487	0.229	4,302
*B. napus*	0.288	0.921	0.313	5,241	0.341	1.563	0.218	4,239
*G. hirsutum*	0.224	0.603	0.371	5,523	-	-	-	82
*C. arabica*	0.229	0.583	0.393	5,508	0.262	0.828	0.316	4,374
*O. europaea*	0.200	0.503	0.398	5,526	0.228	0.708	0.321	4,458
*N. tabacum*	0.213	0.576	0.370	5,592	0.249	0.885	0.281	4,632
*H. annuus*	0.317	0.633	0.502	5,010	0.354	0.799	0.443	4,443
*P. ginseng*	0.223	0.449	0.496	5,487	0.280	0.593	0.472	4,029
*S. indicum*	0.210	0.531	0.396	5,226	0.247	0.742	0.333	4,242

Note: This analysis was performed based on re-annotation of *ycf1* genes using DOGMA; the *ycf1* gene of *P. occidentalis* (No. NC_008335) was selected as the reference gene for *K*a and *K*s estimation.

#### Comparison of evolutionary rates of the 77 genes in the cp genomes between sesame and 13 other plant species

Before examining variation in the evolutionary rates of cp genes, we calculated the *K*a, *K*s and *K*a/*K*s ratio of 77 protein-coding genes in sesame and 13 other dicot species from the asterid and rosid clades (the corresponding genes of *P. occidentalis* were chosen as reference genes) (Figure S6). Results showed that evolutionary rates of cp genes were not uniform. Genes involved in photosynthesis, such as *atpH*, *psaA* and *petN*, evolved more slowly and usually presented low *K*a/*K*s values, while other genes, including *psaI*, involved in photosynthesis, *rpl23*, involved in replication, and *ycf2* and *ycf15* genes with unclear functions, evolved more quickly and had high *K*a/*K*s values (≥0.5).

**Figure 4:**
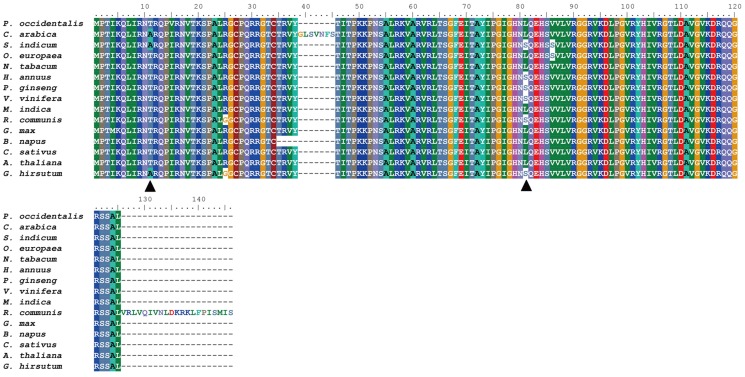
Multiple sequence alignment of *rps12* genes between 15 species. Black triangles indicate amino acids with convergent evolution in both *S. indicum* and *G. hirsutum*.

Comparisons of evolutionary rates of the 77 cp genes between sesame and the other 13 dicot species (Table S1, Figure 4) indicated that nine genes in the sesame cp genome, i.e., the *ndhB*, *ndhD* and *ndhI* genes encoding the subunits of NADH dehydrogenase, the *rpl2*, *rpl22*, *rpl32* and *rpl33* genes encoding the large subunit of the ribosome, the *rps12* gene encoding the small subunit of the ribosome, and the *rbcL* gene encoding the large subunit of Rubisco, all evolved rapidly. Genes with low evolutionary rates included the *ndhK* gene encoding the subunit of NADH dehydrogenase, the *atpI* gene encoding the subunit of ATP synthase, and the *cemA* gene encoding the envelope membrane protein.

## Discussion

In this article, the chloroplast genome of the Chinese cultivar, Yuzhi 11 (white-seeded) was sequenced and the evolutionary characters of cp genome structure and genes were compared between sesame and the 18 species. The marked conservation of the cp genome exists in sesame, and the characteristics of convergent evolution are evident in cp genes in sesame and some other species. To date, more than one hundred cp genomes have been sequenced and studied. Chloroplast genome sequences and basic genomic structures, e.g., gene content, repeat characteristics, and indel and SSR marker locations, have been analyzed in many important crops [18], [30]–[32]. The conservation of the cp genome suggests a universal evolutionary selection pressure; evolutionary changes in the cp genome do not happen randomly [33]. However, in order to clarify plant phylogenic relationships, evolutionary changes in individual species require further exploration.

### Characteristics of the sesame cp genome

With the aid of sesame nuclear genomic data, we have sequenced the cp genome of sesame cv. YuZhi 11 using Illumina and 454 sequencing and explored its species-specific structure. Although recent studies have suggested that the genetic diversity and cytological differences between black-seeded and white-seeded germplasm are significant [34], [35], the cp genome sequence of cv. Yuzhi 11 (white-seeded) has high similarity to that of cv. Ansanggae (black-seeded) (NC_016433.2, with only slight variation in the number of nucleotide repeats in 14 homopolymers which may be to use of different sequencing platforms in these two studies.

The sesame cp genome has a similar number of genes to species such as *Nicotiana tabacum*, *Vitis vinifera* and *Platanus occidentalis*. The order of genes in the sesame cp genome is highly conserved and is similar to that of *N. tabacum*, *A. thaliana*, *P. occidentalis* and *B. napus*, but different from that of *G. max*, *H. annuus* and *G. hirsutum* in which there are large inversions [30], [31], [36]. While gene loss events were not detected, the sesame cp genome has a shortened *ycf2* gene. In addition, some unique repeat sequences, e.g., R13 and R14 (Figure 3) were found, with the number of repeats being lower in sesame than in *A. thaliana*, *G. max* and *G. hirsutum* [30], [31], [37]. IR/SC junctions are located in the *rps19* and *ycf2* genes, respectively, as in some other species.

### Variation in the *ycf1* and *ycf2* gene

*Ycf* genes have proved useful for analyzing cp genome variation in higher plants and algae, even though their function is not thoroughly known [38]. There are 7–8 *ycf* genes (including pseudogenes) in the cp genomes from higher plants. Of these, *ycf1* and *ycf2* are the two largest genes and are located in IR/SC junction and IR region, respectively. Biolistic chloroplast transformation studies in *N. tabacum* have indicated that these genes are essential for plant survival [39] and are likely the targets of positive evolutionary selection [40]. The *ycf2* gene in the cp genome is regarded as having one of the fastest evolutionary rates within the cp genome since one copy of the *ycf2* gene in ginkgo is lost and both copies of the *ycf2* gene in the grasses are lost [40]–[42]. The y*cf2* gene in sesame is transcribed as its mRNA is present in the sesame transcriptome (Zhang H. et al., data not shown), and should thus be functional [43]. In this study, we found that an approximately 1,179 bp fragment of the *ycf2* gene was missing in the sesame cp genome (Figure S3). Moreover, BLAST results showed that querying a 1–585 bp fragment of the *ycf2* gene yielded a hit in the sesame draft genome, however, the remaining 586–1,179 bp fragment was not found (Figure 2B).

Interestingly, multiple sequence alignments showed that a 580–1,179 bp fragment of *ycf*2 in *P. ginseng* and a 439–1155 bp fragment of *ycf*2 in *C. sativus*, counterparts of the 586–1,179 bp query fragment of the sesame *ycf*2 gene, are also missing (Figure 2A, B). We thus propose that this sequence deletion may have occurred in at least one of two ways, i.e., by transfer to the nuclear genome, as in the case of sesame, or by direct deletion, as in the case of *P. ginseng* and *C. sativus*. The evolutionary characteristics of the *ycf2* gene in these species are similar, even though a close phylogenetic relationship was not found between sesame, *P. ginseng* and *C. sativus*, presenting an evident signature of convergent evolution (Figure S2). Similarly, the co-occurred missing event in *ycf1* gene in sesame, *C. arabica* and *B. napus* should be a consequence of convergent evolution (Figure S3).

### Convergent evolution of repeat sequences

Chloroplast genomes in most plants contain repeat sequences other than the Inverted Repeats (IR), with the repeat number ranging from tens to hundreds [30], [44]. Repeat sequences often maintain high conservation of sequence identity and location, and thus may play functional roles in cp genomes [30], [45]. The detailed functions of the repeats are not well understood, though the number of repeats has been shown to be correlated with the degree of rearrangement of the cp genome [46], [47].

R13 and R14, located within the exons of the *ycf2* gene, were found to be unique to the sesame cp genome. Repeats of shorter length are present in the same locations as R13 and R14 in the other species such as *O. europaea*, *V. vinifera* and *G. hirsutum* (Figure 3). The uniqueness of these repeats in sesame is likely to be a consequence of extension of shorter ancestral repeats. Moreover, such conservation of repeats in species that are not phylogenetically closely related should be regarded as an incident of convergent evolution.

### Consequences of IR expansion/contraction

IR expansion and contraction are common evolutionary events in plant species and have been well verified in many species such as *A. thaliana*, *N. tabacum*, and oil palm [37], [48]–[50]. LSC/IR and SSC/IR junctions have different features in the cp genomes of different species. IR expansion/contraction has had two main consequences on the cp genome evolution in almost all publically-available cp genomes, i.e., alteration of cp genome size [30], [51] and formation of pseudogenes at IR/SC junctions. In higher plants, IR expansion/contraction has a major effect on genome size [52], which has also been the case in our study (Table 2). In previous studies, sequences located in IRs showed slower rates of evolution compared with those located in SSC or LSC regions [7]. Here, we also found that the evolutionary rate of the part of the *ycf1* sequences located in the IR region was significantly lower than that of the full sequences in the 13 species (Table 5). Accordingly, we propose that one consequence of IR contraction/expansion is changing the rate of evolution.

### Evolutionary rates suggest convergent evolution

Compared with genes from nuclear genomes, cp genes evolve at a slow rate, making them a useful for plant phylogenetic and taxonomic research [53]. Previous studies have suggested that the evolutionary rate of cp genes is lineage-specific, locus-specific and region-specific [7], [40], [54]. For example, some cp genes in grass lineages have evolved at a faster rate than those from *N. tabacum* [54]; IRs have a slower nucleotide substitution rate compared with SSC and LSC regions [7]. In addition, it has been shown that the rate of evolution of a gene correlates with relaxed or positive selection, gene function, and gene expression level [55], [56]. In the sesame cp genome, the rapid or slow evolution of some genes is species-specific. The evolutionary rate of *rps12* in sesame and *G. hirsutum* is highest in sesame and 13 other species (Table S1). Multiple sequence alignment results suggested that co-variation of two sites in the *rps12* amino acid sequence occurs only in sesame and *G. hirsutum* (Figure 4). Similarly, convergent evolution was also detected in *clpP* genes of sesame and *C. sativus* (Figure S6).

## Conclusion

The cp genome sequence of cv. Yuzhi 11 (white-seeded) has high similarity to that of cv. Ansanggae (black-seeded). The cp gene deletion event occur in cp genomes in at least one of two ways, i.e., transfer to the nuclear genome as has occurred in sesame, and directly deletion as has occurred in *P. ginseng* and *C. sativus*. The uniqueness of repeats in sesame is likely due to extension of shorter ancestral repeats. Apart from changing the cp genome size and forming pseudogenes at IR/SC junctions, changing the rate of evolution is regarded as another new consequence of IR contraction/expansion. The characteristics of convergent evolution are evident in cp genes in sesame and some other species. These findings provide a foundation for further understanding of cp genome evolution in *Sesamum* and other higher plants.

The accession number for the sesame chloroplast genome sequence (cv. Yuzhi 11) is KC569603 (NCBI). The accession number of 454 Roche, 500 bp PE and 3Kb MP *Illumina* sequencing raw data of sesame cp genome is SRR949053, SRR949054 and SRR949055, respectively. The Illumina and Roche 454 raw reads of sesame nuclear genome sequence have been deposited in sesame genome database and could be downloaded from the website of Sesame Genome Project (http://www.sesamum.org).

## Supporting Information

Figure S1 **The methodology of sesame cp genome assembly.**
(TIF)

Figure S2 **Phylogenetic relationship of** ***S. indicum*** **and 18 other plant species based on the NCBI taxonomy database.**
(TIF)

Figure S3 **Multiple sequence alignments of** ***ycf2*** **genes (1–1,200 bp) between 15 species.**
(TIF)

Figure S4 **Multiple sequence alignments of** ***ycf1*** **genes between 15 species.**
(TIF)

Figure S5 **Comparison of the locations of the LSC, IR and SSC border regions between 15 cp genomes.**
(TIF)

Figure S6 **Multiple sequence alignments of** ***clpP*** **gene sequences between 15 species.** Black triangles indicate amino acids with convergent evolution in *S. indicum* and *C. sativus*.
(TIF)

Table S1 **Comparisons of the evolutionary rates of 77 genes between the cp genomes of** ***S. indicum*** **L. and 13 other species.** All 77 genes were re-annotated using DOGMA; - indicates that no such gene exists in that species, or the gene cannot be estimated using the MA method; * indicates *K*a/*K*s values larger than 2 which are not credible due to their low *K*s values.
(XLS)

## References

[1] Ashri A (1998) Sesame Breeding. In: Janick J, editor. Plant Breeding Reviews. Oxford: Oxford Press. pp. 79–228.

[2] Bedigian D, Harlan JR (1986) Evidence for cultivation of sesame in the ancient world. Econ Bot 40: 137–154.

[3] Arslan C, Uzun B, Ülger S, Çağırgan Mİ (2007) Determination of oil content and fatty acid composition of sesame mutants suited for intensive management conditions. J Am Oil Chem Soc 84: 917–920.

[4] Nakimi M (1995) The chemistry and physiological functions of sesame. Food Rev Int 11: 281–329.

[5] Anilakumar KR, Pal A, Khanum F, Bawa AS (2010) Nutritional, medicinal and industrial uses of sesame (*Sesamum indicum* L.) seeds- an overview. Agric Conspec Sci 75: 159–168.

[6] Zhang H, Miao H, Wang L, Qu L, Liu H, et al (2013) Genome sequencing of the important oilseed crop *Sesamum indicum* L. Genome Biol. 14: 401. 10.1186/gb-2013-14-1-401 PMC3663098 23369264

[7] Yi DK, Kim KJ (2012) Complete chloroplast genome sequences of important oilseed crop *Sesamum indicum* L. PLoS ONE 7: e35872. 22606240 10.1371/journal.pone.0035872 PMC3351433

[8] Nimmakayala P, Perumal R, Mulpuri S, Reddy UK (2011) Sesamum. In: Kole C, editor. Wild corp relatives: genomic and breeding resources oilseeds. Berlin: Springer-Verlag Press. pp. 261–273.

[9] Sugiura M (2003) History of Chloroplast genomics. Photosynthetic Res76: 371–377. 10.1023/A:1024913304263 16228593

[10] Xiong AS, Peng RH, Zhuang J, Gao F, Zhu B, et al (2009) Gene duplication, transfer, and evolution in the chloroplast genome. Biotechnol Adv 27: 340–347. 19472510 10.1016/j.biotechadv.2009.01.012

[11] Sugiura M (1989) The chloroplast chromosomes in land plants. Annu Rev Cell Biol 5: 51–70. 2688709 10.1146/annurev.cb.05.110189.000411

[12] Olmstead RG, Palmer JD (1994) Chloroplast DNA systematic: A review of methods and data analysis. Am J Bot 81: 1205–1224.

[13] Chumley TW, Palmer JD, Mower JP, Fourcade HM, Calie PJ, et al (2006) The complete chloroplast genome sequence of *Pelargonium* × *hortorum*: organization and evolution of the largest and most highly rearranged chloroplast genome of land plants. Mol Biol Evol 23: 2175–2190. 16916942 10.1093/molbev/msl089

[14] Corriveau JL, Coleman AW (1988) Rapid screening method to detect potential biparental inheritance of plastid DNA and results for over 200 angiosperms. Am J Bot 75: 1443–1458.

[15] Säll T, Jakobsson M, Lind-Halldén C, Halldén C (2003) Chloroplast DNA indicates a single origin of the allotetraploid *Arabidopsis suecica* . J Evol Biol 16: 1019–1029. 14635917 10.1046/j.1420-9101.2003.00554.x

[16] Ruf S, Karcher D, Bock R (2007) Determining the transgene containment level provided by chloroplast transformation. Proc Natl Acad Sci U S A 104: 6998–7002. 17420459 10.1073/pnas.0700008104 PMC1849964

[17] Saski C, Lee SB, Fjellheim S, Guda C, Jansen RK, et al (2007) Complete chloroplast genome sequences of *Hordeum vulgare*, *Sorghum bicolor* and *Agrostis stolonifera*, and comparative analyses with other grass genomes. Theor Appl Genet 115: 571–590. 17534593 10.1007/s00122-007-0567-4 PMC2674615

[18] Young HA, Lanzatella CL, Sarath G, Tobias CM (2011) Chloroplast genome variation in upland and lowland switchgrass. PLoS ONE 6: e23980 doi:10.1371/journal.pone.0023980 21887356 10.1371/journal.pone.0023980 PMC3161095

[19] Zhang T, Zhang H, Wei S, Zheng Y, Zhang Z, et al (2003) Analysis of Integrated characteristics of Yuzhi 11. Chinese Agri Sci Bullet 19: 44–46.

[20] Oharamays EP, Capwell JC (1993) Miniprep for chloroplast DNA isolation. Microchem J 47: 245–250.

[21] Jarvie T, Harkins T (2008) 3K Long-Tag Paired End sequencing with the Genome Sequencer FLX System. Nat Methods 5: 1–2. 18476839 10.2144/000112894

[22] Cox M, Peterson D, Biggs P (2010) SolexaQA: At-a-glance quality assessment of Illumina second-generation sequencing data. BMC bioinformatics 11: 485. 20875133 10.1186/1471-2105-11-485 PMC2956736

[23] Li H, Durbin R (2010) Fast and accurate long-read alignment with Burrows-Wheeler transform. Bioinformatics 26: 589–595. 20080505 10.1093/bioinformatics/btp698 PMC2828108

[24] Zerbino DR, Birney E (2008) Velvet: algorithms for de novo short read assembly using de bruijn graphs. Genome Res 18: 821–829. 18349386 10.1101/gr.074492.107 PMC2336801

[25] Wyman SK, Jansen RK, Boore JL (2004) Automatic annotation of organellar genomes with DOGMA. Bioinformatics 20: 3252–3255. 15180927 10.1093/bioinformatics/bth352

[26] Krzywinski M, Schein J, Birol I, Connors J, Gascoyne R, et al (2009) Circos: an information aesthetic for comparative genomics. Genome Res 19: 1639–1645. 19541911 10.1101/gr.092759.109 PMC2752132

[27] Kurtz S, Choudhuri JV, Ohlebusch E, Schleiermacher C, Stoye J, et al (2001) REPuter: the manifold applications of repeat analysis on a genomic scale. Nucleic Acids Res 29: 4633–4642. 11713313 10.1093/nar/29.22.4633 PMC92531

[28] Tamura K, Peterson D, Peterson N, Stecher G, Nei M, et al (2011) MEGA5: Molecular evolutionary genetics analysis using maximum likelihood, evolutionary distance, and maximum parsimony methods. Mol Biol Evol 28: 2731–2739. 21546353 10.1093/molbev/msr121 PMC3203626

[29] Zhang Z, Li J, Zhao XQ, Wang J, Wong GK, et al (2006) KaKs_Calculator: calculating *K*a and *K*s through model selection and model averaging. Genomics Proteomics Bioinformatics 4: 259–263. 17531802 10.1016/S1672-0229(07)60007-2 PMC5054075

[30] Saski C, Lee SB, Daniell H, Wood TC, Tomkins J, et al (2005) Complete chloroplast genome sequence of *Gycine max* and comparative analyses with other legume genomes. Plant Mol Biol 59: 309–322. 16247559 10.1007/s11103-005-8882-0

[31] Lee SB, Kaittanis C, Jansen RK, Hostetler JB, Tallon LJ, et al (2006) The complete chloroplast genome sequence of *Gossypium hirsutum*: organization and phylogenetic relationships to other angiosperms. BMC Genomics 7: 61. 16553962 10.1186/1471-2164-7-61 PMC1513215

[32] Yi DK, Lee HL, Sun BY, Chung MY, Kim KJ (2012) The complete chloroplast DNA sequence of *Eleutherococcus senticosus* (Araliaceae); comparative evolutionary analyses with other three asterids. Mol Cells 33: 497–508. 22555800 10.1007/s10059-012-2281-6 PMC3887725

[33] Bungard RA (2004) Photosynthetic evolution in parasitic plants: insight from the chloroplast genome. BioEssays 26: 235–247. 14988925 10.1002/bies.10405

[34] Yue W, Wei L, Zhan T, Li C, Miao H, et al (2012) Analysis of genetic diversity and population structure of germplasm resources in sesame (*Sesamum indicum* L.) by SSR markers. Acta Agronomica Sinica 38: 2286–2296.

[35] Zhang H, Miao H, Li C, Wei L, Ma Q (2012) Analysis of Sesame Karyotype and Resemblance-near Coefficient. Chinese Plant Bullet 47: 602–614.

[36] Timme RE, Kuehl JV, Boore JL, Jansen RK (2007) A comparative analysis of the *Lactuca* and *Helianthus* (*Asteraceae*) plastid genomes: identification of divergent regions and categorization of shared repeats. Am J Bot 94: 302–312. 21636403 10.3732/ajb.94.3.302

[37] Sato S, Nakamura Y, Kaneko T, Asamizu E, Tabata S (1999) Complete Structure of the Chloroplast Genome of *Arabidopsis thaliana* . DNA Res 6: 283–290. 10574454 10.1093/dnares/6.5.283

[38] Stoebe B, Martin W, Kowallik KV (1998) Distribution and nomenclature of protein-coding genes in 12 sequenced chloroplast genomes. Plant Mol Biol Rep 16: 243–255.

[39] Drescher A, Ruf S, Calsa TJ, Carrer H, Bock R (2000) The two largest chloroplast genome-encoded open reading frames of higher plants are essential genes. Plant J 22: 97–104. 10792825 10.1046/j.1365-313x.2000.00722.x

[40] Parks MB (2011) Plastome phylogenomics in the genus Pinus using massively parallel sequencing technology. PhD thesis. Oregon State University, Botany and Plant Pathology Department.

[41] Diekmann K, Hodkinson TR, Wolfe KH, van den Bekerom R, Dix PJ, et al (2009) Complete chloroplast genome sequence of a major allogamous forage species, perennial ryegrass (*Lolium perenne* L.). DNA Res 16: 165–176. 19414502 10.1093/dnares/dsp008 PMC2695775

[42] Lin CP, Wu CS, Huang YY, Chaw SM (2012) The complete chloroplast genome of Ginkgo biloba reveals the mechanism of inverted repeat contraction. Genome Biol Evol 4: 374–381. 22403032 10.1093/gbe/evs021 PMC3318433

[43] Zhang H, Wei L, Miao H, Zhang T, Wang C (2012) Development and validation of genic-SSR markers in sesame by RNA-seq. BMC Genomics 13: 316. 22800194 10.1186/1471-2164-13-316 PMC3428654

[44] Mariotti R, Cultrera NG, Díez CM, Baldoni L, Rubini A (2010) Identification of new polymorphic regions and differentiation of cultivated olives (*Olea europaea* L.) through plastome sequence comparison. BMC Plant Biol 10: 211. 20868482 10.1186/1471-2229-10-211 PMC2956560

[45] Daniell H, Lee SB, Grevich J, Saski C, Quesada-Vargas T, et al (2006) Complete chloroplast genome sequences of *Solanum bulbocastanum*, *Solanum lycopersicum* and comparative analyses with other Solanaceae genomes. Theor Appl Genet 112: 1503–1518. 16575560 10.1007/s00122-006-0254-x

[46] Pombert JF, Otis C, Lemieux C, Turmel M (2005) The chloroplast genome sequence of the green alga *Pseudendoclonium akinetum Ulvophyceae* reveals unusual structural features and new insightsinto the branching order of chlorophyte lineages. Mol Biol Evol 22: 1903–1918. 15930151 10.1093/molbev/msi182

[47] Haberle RC, Fourcade HM, Boore JL, Jansen RK (2008) Extensive rearrangements in the chloroplast genome of *Trachelium caeruleum* are associated with repeats and tRNA genes. J Mol Evol 66: 350–61. 18330485 10.1007/s00239-008-9086-4

[48] Shinozaki K, Ohme M, Tanaka M, Wakasugi T, Hayashida N, et al (1986) The complete nucleotide sequence of the tobacco chloroplast genome: its gene organization and expression. EMBO J 5: 2043–2049. 16453699 10.1002/j.1460-2075.1986.tb04464.x PMC1167080

[49] Kim KJ, Lee HL (2004) Complete chloroplast genome sequences from Korean ginseng (*Panax schinseng* Nees) and comparative analysis of sequence evolution among 17 vascular plants. DNA Res 11: 247–261. 15500250 10.1093/dnares/11.4.247

[50] Uthaipaisanwong P, Chanprasert J, Shearman JR, Sangsrakru D, Yoocha T, et al (2012) Characterization of the chloroplast genome sequence of oil palm (*Elaeis guineensis* Jacq.). Gene 500: 172–180. 22487870 10.1016/j.gene.2012.03.061

[51] Wolf PG, Der JP, Duffy AM, Davidson JB, Grusz AL, et al (2011) The evolution of chloroplast genes and genomes in ferns. Plant Mol Biol 76: 251–261. 20976559 10.1007/s11103-010-9706-4

[52] Ibrahim RI, Azuma J, Sakamoto M (2006) Complete nucleotide sequence of the cotton (*Gossypium barbadense* L.) chloroplast genome with a comparative analysis of sequences among 9 dicot plants. Genes Genet Syst 81: 311–321. 17159292 10.1266/ggs.81.311

[53] Khan A, Khan IA, Asif H, Azim MK (2010) Current trends in chloroplast genome research. Afr J Biotechnol 9: 3494–3500.

[54] Gaut BS, Muse SV, Clegg MT (1993) Relative rates of nucleotide substitution in the chloroplast genome. Mol Phylogenet Evol 2: 89–96. 8043149 10.1006/mpev.1993.1009

[55] McInerney JO (2006) The causes of protein evolutionary rate variation. Trends Ecol Evol 21: 230–232. 16697908 10.1016/j.tree.2006.03.008

[56] Wang Z, Zhang J (2009) Why is the correlation between gene importance and gene evolutionary rate so weak? PLoS Genet 5: e1000329. 19132081 10.1371/journal.pgen.1000329 PMC2605560

