# Supplementary figures and images for: Insights from the Complete Chloroplast Genome into the Evolution of Sesamum indicum L
Source: PLoS One. 2013 Nov 26;8(11):e80508. doi: 10.1371/journal.pone.0080508 (PMC3841184; doi:10.1371/journal.pone.0080508)

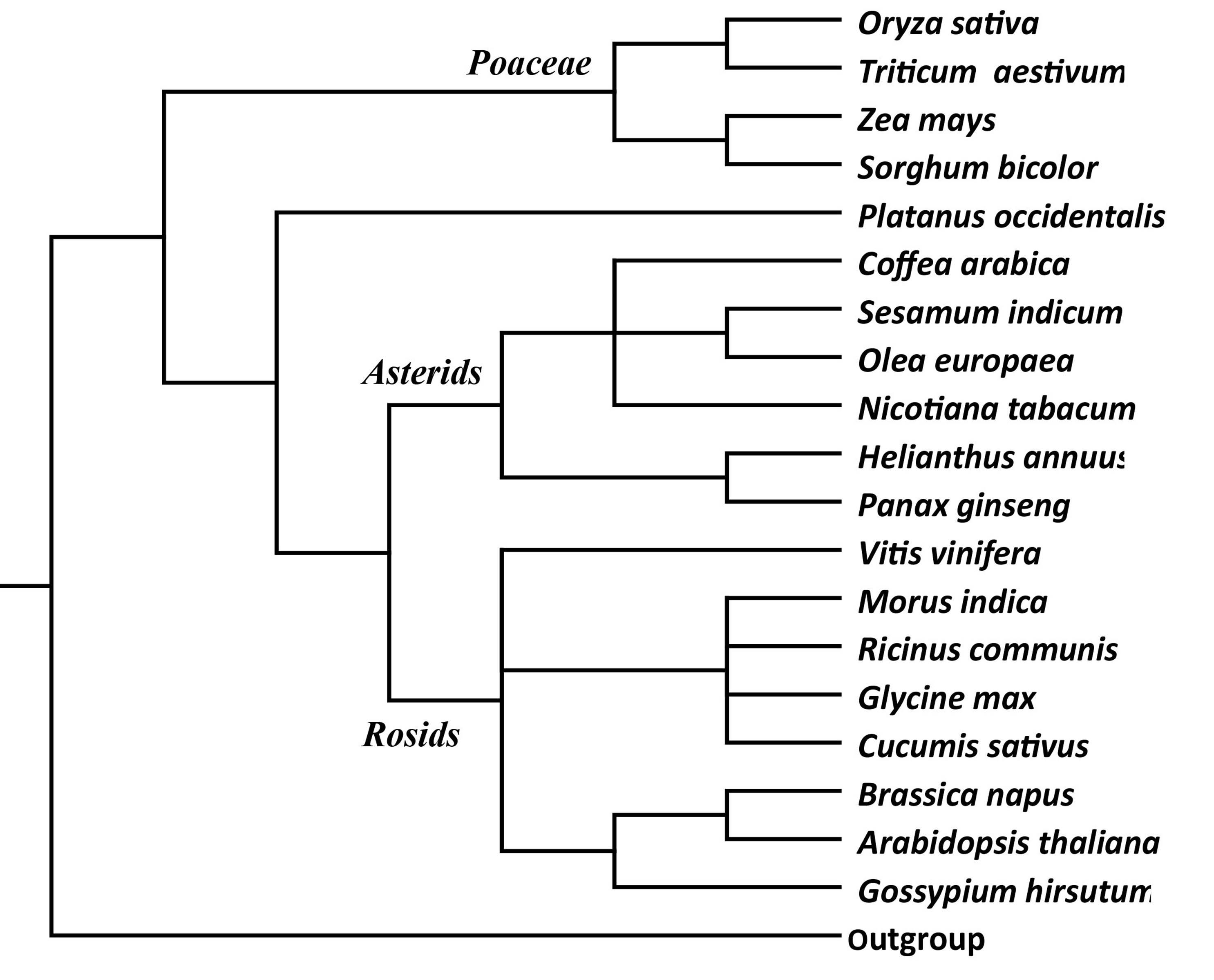

Supplement: Figure S2 — Phylogenetic relationship of S. indicum and 18 other plant species based on the NCBI taxonomy database. (TIF) [file pone.0080508.s002.tif]

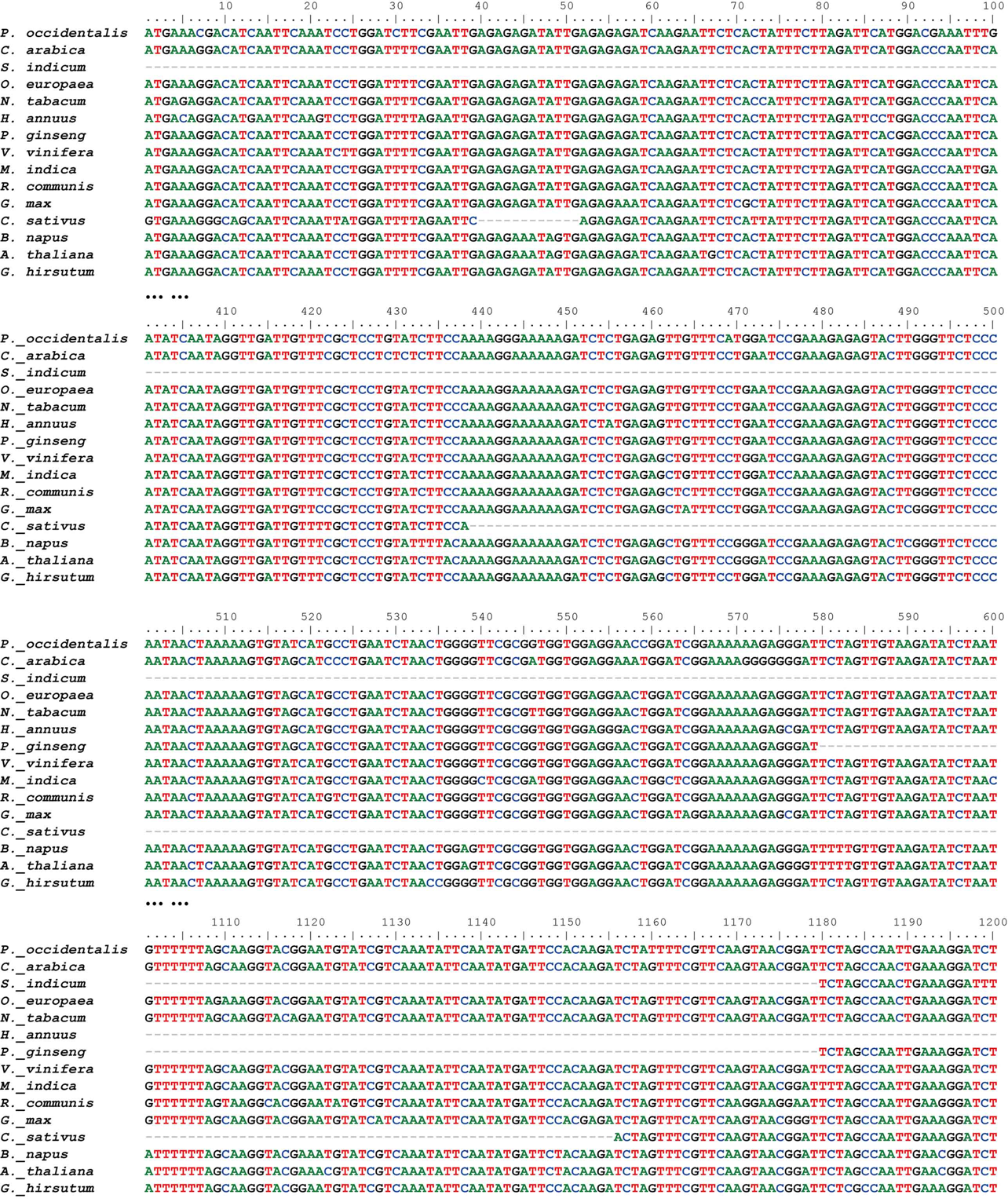

Supplement: Figure S3 — Multiple sequence alignments of ycf2 genes (1–1,200 bp) between 15 species. (TIF) [file pone.0080508.s003.tif]

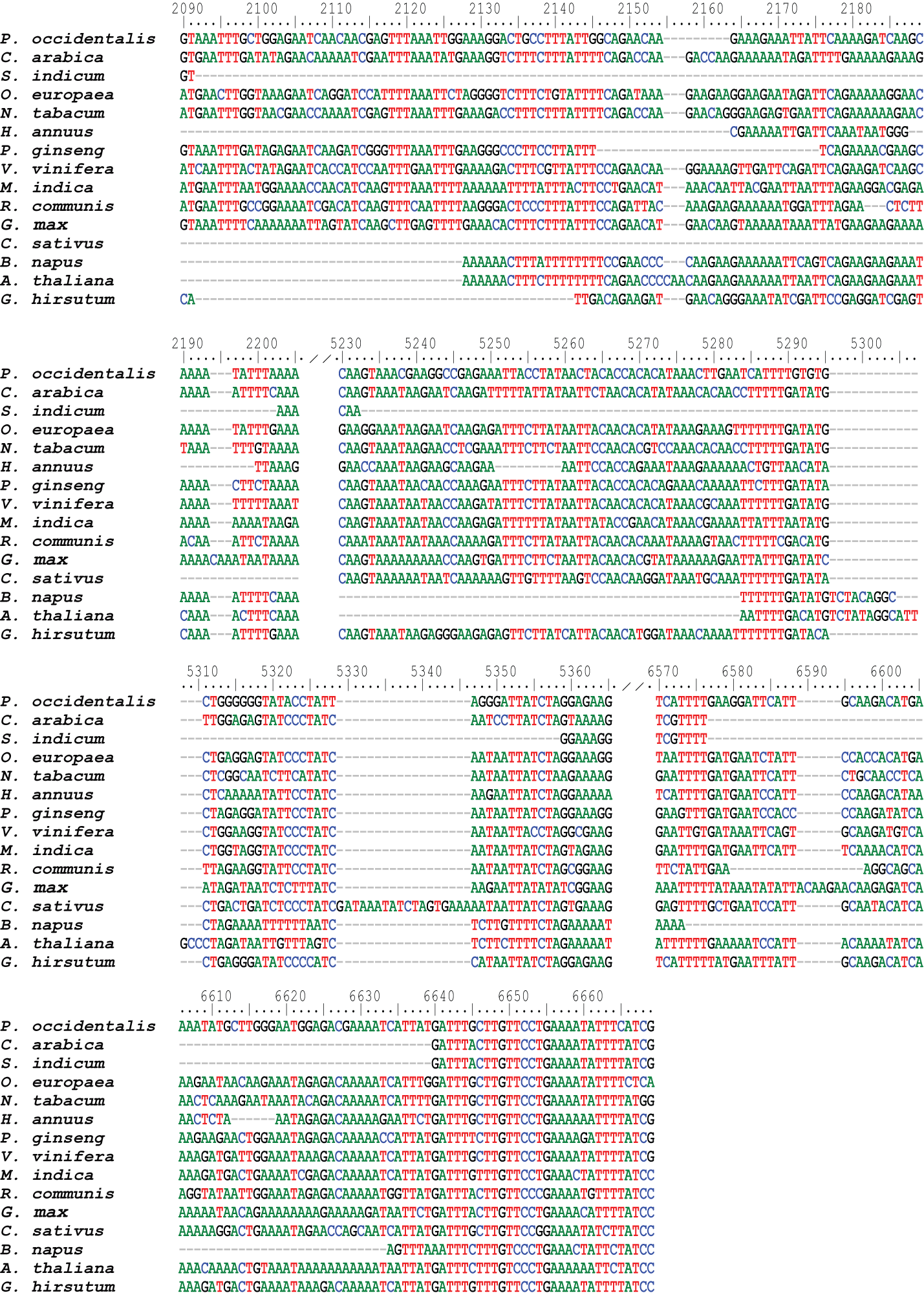

Supplement: Figure S4 — Multiple sequence alignments of ycf1 genes between 15 species. (TIF) [file pone.0080508.s004.tif]

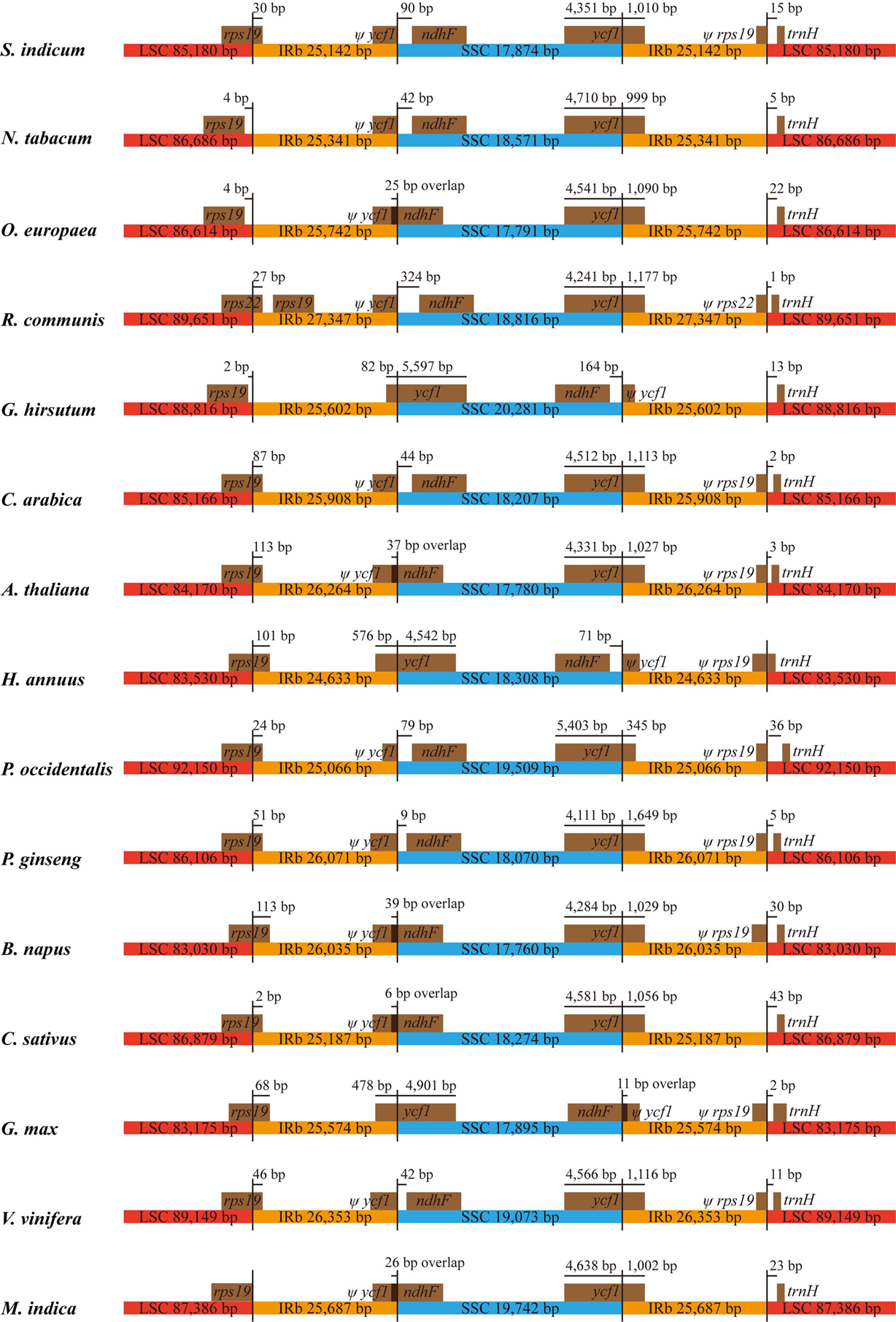

Supplement: Figure S5 — Comparison of the locations of the LSC, IR and SSC border regions between 15 cp genomes. (TIF) [file pone.0080508.s005.tif]

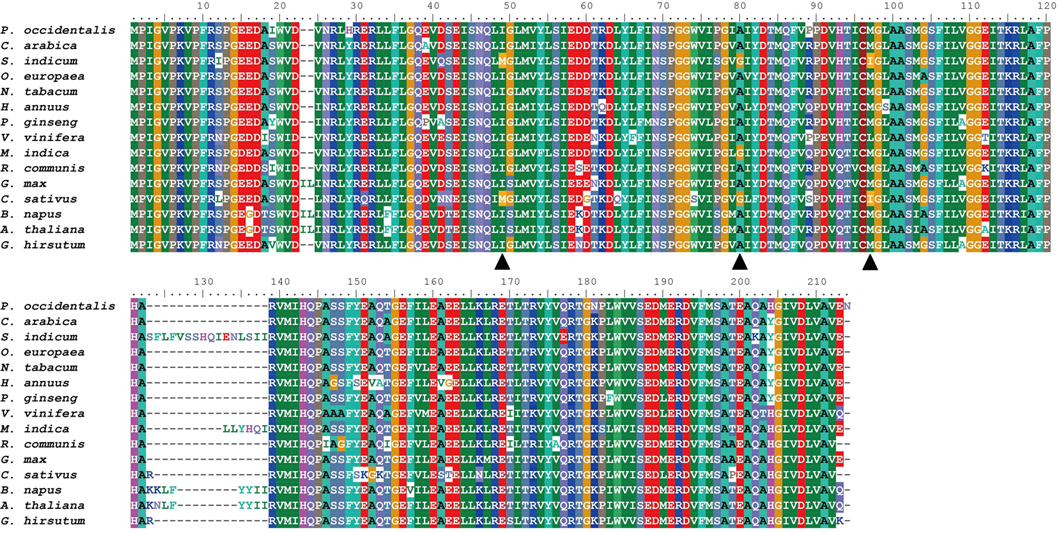

Supplement: Figure S6 — Multiple sequence alignments of clpP gene sequences between 15 species. Black triangles indicate amino acids with convergent evolution in S. indicum and C. sativus. (TIF) [file pone.0080508.s006.tif]
